# Research analytics on the applications of flexible formwork gob-side entry retention technology in medium-thickness coal seams with large inclination angles

**DOI:** 10.1371/journal.pone.0323337

**Published:** 2025-05-12

**Authors:** Xiaofan Cao, Song Wang, Xiaoli Wang, Zhongyu Yang, Ang Li

**Affiliations:** 1 School of Architecture and Civil Engineering, Xi ‘an University of Science and Technology, Xi’an, Shaanxi, China; 2 School of Management, Xi ‘an University of Science and Technology, Xi’an, Shaanxi, China; 3 Shaanxi Pioneer Construction Technology Co., Ltd, Xi’an, Shaanxi, China; China Construction Fourth Engineering Division Corp. Ltd, CHINA

## Abstract

Gob-side entry retention (GER) has been successfully advanced and implemented for several years. Notably, strong economic returns have been realised for different thicknesses of coal seams under near-horizontal and gently inclined conditions. However, implementing gob-side entry retention under the conditions of high dip angle or steeply inclined coal seam has always been difficult. This paper systematically introduces the material and construction processes of flexible formwork GER. Engineering practice, theoretical calculations and indoor test analysis were used to propose an independently designed and developed special-shaped flexible formwork concrete wall with a width of 1.2 m, a strength of C30 and an inclination angle of 20°. This wall was then used to investigate the GER technology in the No. 31342 working face of the Dachiba Coal Mine. The findings reveal that the strength of the developed coal solid waste flexible formwork concrete fulfils the design requirements, and the residual strength and healing capacity are superior to that of ordinary concrete. The design of the supporting parameters of flexible formwork GER was satisfactory in the No. 31342 working face of the Daichiba Coal Mine. The overall deformation of the roadway is small, and a decent effect is obtained with respect to retaining the roadway. Hence, the technical challenge of enacting GER in medium-thick coal seams with a high dip angle under the filling and supporting modes has been resolved successfully.

## 1 Introduction

Coal is a crucial component of China’s energy structure. Considering the present trajectory, China’s coal consumption will surpass more than 50% of the total energy consumption by 2030 and continue to do so by more than 35% by 2050 [1–6]. In recent years, with the gradual depletion of high-quality coal resources and shrinking production in eastern China, the focus of resource development has been shifted to the western region [7]. Most of these areas have inclined coal seams, accounting for more than 60%, and generally exhibit a high mining value. However, in the process of mining inclined coal seams, the efficiency of coal mining is substantially reduced because of the inclination angle of the roadway and the asymmetry of the roadway surrounding the rock stress [8–10].

**Fig 1:**
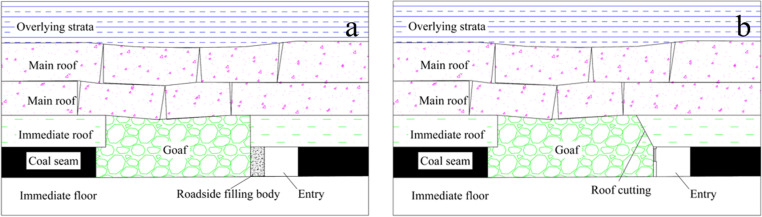
Support mode of GER. (a) Filling support; (b) Roof-cutting support.

GER technology is currently the most popular method to enhance the recovery rate of coal resources [11–19]. Based on the various roadway support methods, roadway support can be divided into filling support and roof-cutting support (Fig 1). Filling support refers to the filling and pouring of a continuous wall on the side of the roadway next to the mining region to isolate the mining area, the most representative of which is the flexible formwork gob-side entry retention (FFGER). FFGER has been successfully applied in near-horizontal and gently inclined coal seams with various thicknesses, and considerable economic and social benefits have been achieved because of its applications [15,20–27].

The abovementioned findings obtained from research works have perfected and advanced the theory and technology of FFGER to a great extent. However, investigations on the theory and technique of inclined coal seams still lag far behind actual production requirements. This drawback prevents the safe and efficient production of inclined coal seams, especially the coal seam with a large inclination angle (35° ~ 55°) and sharp inclination (>55°) [28]. For instance, in inclined coal seams, a cantilever beam at the immediate roof strata is likely to slip in the inclination direction, exerting lateral pressure on the roadside support body [29–31].

There are a few applicable GER technology reports for coal seams with large inclination angles. However, this execution has not been successfully promoted. According to the status quo of the applications of the technology of GER in large inclination coal seams, this paper considers the working face of No. 31342 in the large inclination medium-thickness coal seam of Daichiba Coal Mine of the Sichuan Coal Group as the research background. The authors have conducted theoretical and practical research on the FFGER in the middle-thickness coal seam with a large inclination angle for the first time in this industry. The theoretical and experimental studies presented in this paper can overcome the challenges in mine production and provide a reference for the expansion and applications of FFGER for coal seams with large inclination or even sharp inclination.

## 2 Introduction to FFGER

### 2.1 Flexible formwork concrete

After pumping, the concrete wall of the bound flexible formwork held through pulling anchor bolts has an initial load capacity of 0.8 MPa. By applying flexible formwork concrete, one can resolve the technical issue of the inability to pour continuous mass structure concrete in underground engineering.

The length of each flexible formwork can be adjusted according to the construction conditions; the common formwork length is 3 m. The formwork’s height is greater than that of the pouring space, and the general surplus is 0.3 m. The formwork’s thickness is the thickness of the roadside filling body, which is determined by theoretical calculation and the construction conditions.

#### 2.1.1 The function of flexible form wall.

As coal mining operations continue to forge ahead, the integrity and stability of the flexible formwork wall are further enhanced with the increased age of the concrete, and the flexible formwork concrete becomes considerably more restricted horizontally.

With the continuous progress of the working face, the flexible formwork wall must have a higher initial bracing force and faster resistance to upgrade the performance. However, the growth of the wall strength requires a certain amount of time and space, necessitating the use of equipment and walls to generate cooperative support within an appropriate range.

The equipment used at this stage includes gangue blocking brackets, hanging mould brackets, unit brackets, special end brackets and monoliths. They work in tandem with the flexible formwork walls to increase resistance and carry the top slab loads, effectively controlling the delamination rate and rotational deformation of the top slab before concrete curing.

#### 2.1.2 Stability calculation of flexible formwork wall.

Because of the large inclination angle of the working face, the bearing capacity of the flexible form wall adjacent to the roadway and its stability must be considered so that it will not be squeezed by the gangue in the goaf and will slip and overturn. Therefore, the flexible wall’s stability must be considered and checked. During the checking calculation, it is assumed that the side pressure of the goaf reaches the maximum.

- Calculation of the lateral pressure of gangue [32]

$$E_{a}=\frac{1}{2}\gamma {H_{1}}^{2}\tan^{2}(45^{\circ }-\frac{\varphi }{2})$$ (1)

where *E*_*a*_ is the gangue pressure on the wall, kN/m^2^; *γ* is the loose gangue bulk weight in the extraction zone, kN/m^3^; *H*_1_ is the height of the scattered gangue, m; and *φ* is the angle of internal friction of the scattered gangue, °.

- Calculation of overturning stability

$$K_{t}=\frac{G\times a+E_{a}\sin\alpha \times b}{E_{a}\cos\alpha \times z}$$ (2)

where *K*_t_ is the factor of safety against overturning, with a minimum value of 1.5; *G* is the self-weight of the support per linear metre, kN/m; *a* is the distance of the centre of support weight from the point of instability of the support body, m; *b* is the distance of the gangue’s lateral pressure from the instability point of the support body, m; *α* is the angle of inclination of the coal seam being mined, º; *z* is the distance between the action point of the gangue lateral pressure and the instability point of the concrete support body, m. When *K*_t_ is greater than the minimum value of 1.5, it can be inferred that the roadside support wall will not overturn.

- Calculation of sliding stability

The sliding stability check is represented in the following equation:


$$K_{a}=\frac{(G+E_{a}\sin\alpha )\mu }{E_{a}\cos\alpha }$$ (3)

where *K*_*a*_ is the coefficient of safety against skidding with a minimum value of 1.6; *μ* is the friction factor between the support and the contact surface. When *K*_*a*_ is greater than the minimum value of 1.6, it is considered that the slip will not occur.

### 2.2 Technology and Process of FFGER

**Fig 2:**
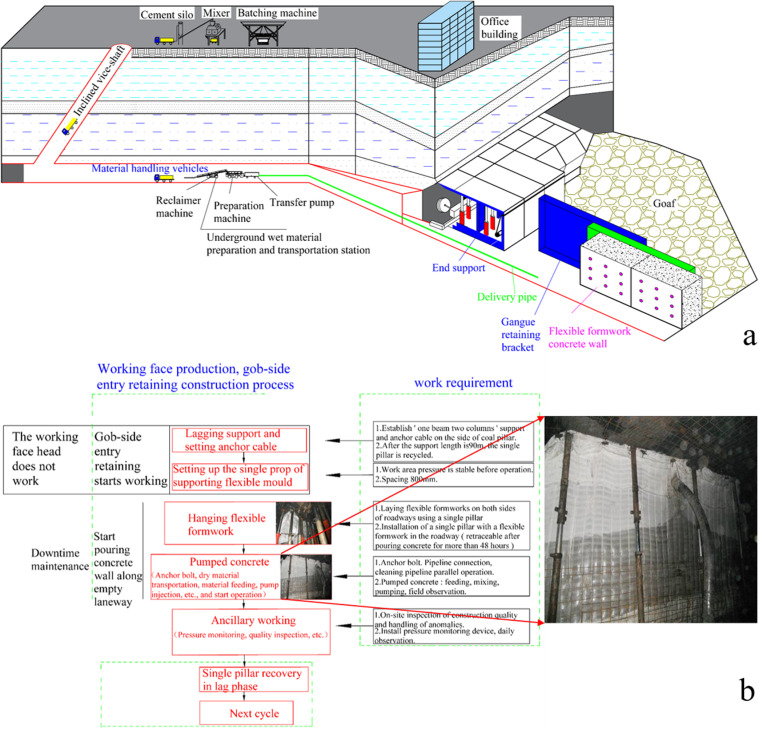
FFGER. (a) Technical process; (b) Operational procedure.

The construction technology of FFGER includes several crucial steps, such as the preparation of dry mixture, the transportation of dry mixture and the creation of concrete mixture underground (Fig 2a). The construction process primarily includes the strengthening support for gob-side entry retaining, concrete preparation and flexible support (Fig 2b).

## 3 Engineering overview

### 3.1 Engineering background

The Daichiba Coal Mine is located in Guangyuan City, Sichuan Province, China. It belongs to the Guangwang Energy Development Limited Liability Company of Sichuan Coal Industry Group Limited. The coal seams mined by Guangwang Energy Development Co., Ltd. are predominately large dip and sharply inclined seams with large variations in thickness, generally at a depth of more than 350 m. The direct roof is thin, with a strength of less than medium hardness, which belongs to the medium integrity roof.

In recent years, the Sichuan Coal Group has made several attempts and innovations in non-pillar mining technology and has achieved positive results. The primary filling methods used for GER in this group are the gangue bag, ordinary concrete wall, paste filling and so on. These techniques are mostly adapted to angles below 25° and coal seams with thicknesses below 2.5 m. Table 1 shows the statistics of some of their results. Therefore, for the Sichuan Coal Group, it is necessary to examine the GER technology at high-angle and medium-thick coal seams and gradually extend it to a steeply inclined coal seam.

**Table 1:** Statistics of gob-side roadway retention in Sichuan Coal Group.

Roadway support mode	Dip angle/ °	Coal seam thickness/ m	Roof lithology
Gangue bag	＜25	1-2.5	Siltstone, coarse sandstone, medium sandstone, mudstone
Concrete wall	＜20	0.5-2,4	Limestone, siltstone, mudstone, medium sandstone
Paste filling	＜10	＜2.5,3-4.5	Limestone, siltstone, mudstone, mudstone, muddy sandstone
Top cutting	＜14	＜2.5,2.5-3.5	Limestone, siltstone, mudstone
building block	＜10	＜2.5	siltstone, mudstone

### 3.2 Introduction to the working face

**Fig 3:**
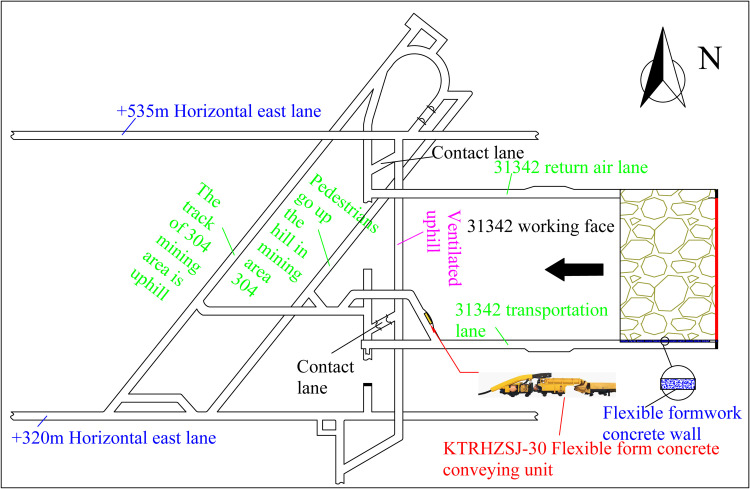
Layout of working face.

The Daichiba Coal Mine has adopted the “S” type gangue net flexible roadway protection technology in the No. 31343 fully mechanised mining face. This mine has employed roadway protection. The GER is adopted while using the transportation roadway of the No. 31342 working face as the background (Fig 3). The return air roadway of the No. 31342 working face has been excavated, and the transport roadway is being arranged. The lower No. 31344 transport roadway is not arranged, and the mining of the No. 31343 working face in the west has been completed.

**Fig 4:**
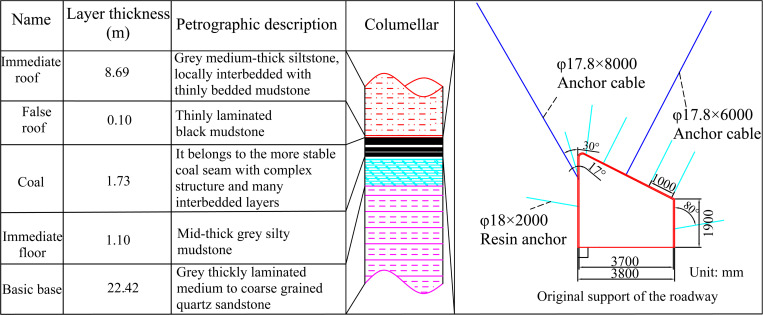
Engineering geological conditions.

The No. 31342 working face is located at level 304 of the mining area at an altitude of + 320 m. Importantly, the mining elevation is + 525 m– + 425 m, the average inclination length of the working face is 155 m, and the strike length is 900 m. The average buried depth of the working face is more than 500 m, and the ground stress is about 13.0 MPa. 13# The coal seam structure is complicated, accompanied by several gangue layers and hard coal quality. The coal seam is a monoclinic structure with a strike close to the east-west. The dip angle of the coal seam does not change much, and it is generally 35° ~ 42°, with an average value of 39°. The average thickness of the coal seam is 1.73 m, and the maximum thickness is 2.2 m, which is the value for an unstable coal seam. The immediate roof is grey thin-layer siltstone and silt-sandy mudstone, and the immediate floor is grey thin-layer silt-sandy mudstone. A 0.1–0.25 m mud-containing coal-debris pseudo-roof is at the top of local coal seams. The average thickness of the central roof of the working face is 8.69 m, and the roof plate is hard and has good integrity. Fig 4illustrates the comprehensive column chart of the coal seam.

The anchor mesh cable supports the roadway of the No. 31342 working face. The roof anchor cable specification is *φ* = 17.8 × 6000 mm, the specification of the upper-side anchor cable is *φ* = 17.8 × 8000 mm, and the anchor cable spacing is 3000 mm. Anchor specifications are detailed as follows: Resin bolts of *φ* = 18 × 2000 mm are used at the top and helpers, and the row spacing between the roof anchors is 1000 × 1500 mm. There are five bolts in a row. When the anchor cable coincides with the anchor rod, the anchor cable is used to protect the roof. The row distance of the anchor rod on the side is 1500 mm, the upper bolt is 800 mm from the roof, the lower bolt is 1600 mm from the bottom plate, and the lower side is 700 mm from the bottom plate. The rhombic metal mesh is used to protect the roof of the whole section (Figure 4).

### 3.3 Calculation and model

- Mechanical Models and Structures

**Fig 5:**
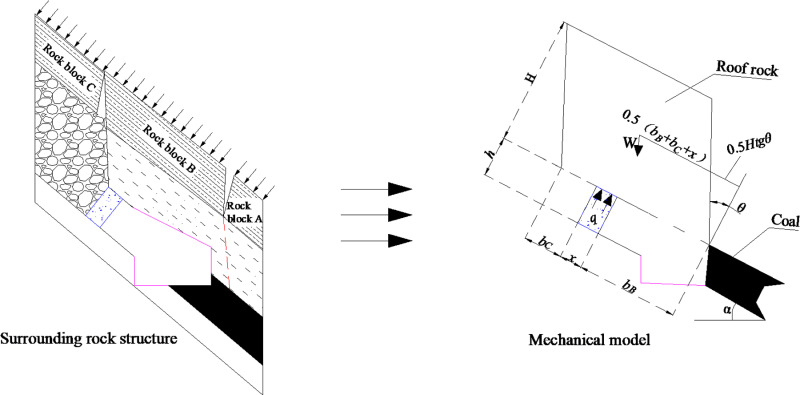
Structure and mechanical model of surrounding rock.

The strength, geometry and structure of the flexible formwork wall determine the success of GER. After combining the actual situation of the No. 31342 working face in the Daichiba Coal Mine, the surrounding rock structure and mechanical model shown in Fig 5 can be generated.

The figure shows that the mechanical model is the separated rock block method model [23]. Generally, *H* = 8*h*, and θ = 26°.


$$q=\frac{8h\tan\theta +2(b_{B}+x+b_{C})}{x}\times \frac{h(b_{B}+x+b_{C})\gamma_{s}\times \cos\alpha }{b_{B}+0.5x}$$ (4)

where *q* is the support load, kN/m^2^; *b*_B_ is the spacing between the inner wall of the flexible wall and the coal wall, m; *x* is the thickness of the flexible wall, m; *b*_C_ is the cantilever on the side of the mining area, m; *γ*_S_ is the bulk weight of the rock mass, kN/m^3^; *H* is the thickness of the coal mine, m; *θ* is the shear angle, generally having the empirical value of 26°, and *α* is the inclination of the coal seam being mined, °.

- Calculation of support capacity of the flexible wall [33]

$$N=0.9\varphi_{1}(f_{c}+4\sigma_{r})A_{c}^{\prime }$$ (5)

The expression of *σ*_*r*_ in the formula is described as follows:


$$\sigma_{r}=\frac{\pi d^{2}\cdot \sigma_{b}}{4a_{1}\cdot a_{2}}$$ (6)

where *N* is the support force of the support body; *φ*_1_ is the stability factor of the support; *f*_*c*_ is the design value of the axial compressive strength of the support, MPa; *σ*_*r*_ is the effective binding force produced by the action of the anchor bolt ferrule, MPa; *A*_*c*_’ is the cross-sectional area, m^2^; *d* is the diameter of the anchor bolt, mm; *σ*_*b*_ is the design value of the tensile strength of the anchor bolt, MPa; and *a*_1_ and *a*_2_ are the spacing and row spacing of the anchor bolts, respectively, mm.

## 4 Design and practice

### 4.1 Calculation of relevant parameters

- Calculation of support and bearing capacity

According to the on-the-spot investigation, the relevant parameters of formulas (4)~(6) can be obtained as follows: *b*_B _= 4.4 m, *x* = 0.8 ~ 1.4 m, *b*_C _= 12.8 m, γ_S _= 24 kN/m^3^, *h* = 2.2 m, θ = 26° and α = 42°. According to the calculation, when the thickness of the flexible wall is 0.8, 1.0, 1.2, and 1.4 and the length direction is 1 m, the supporting resistance of the flexible wall is 19,570 kN, 15,623.5 kN, 12,995.6 kN and 11,122 kN, respectively. The design values of the axial compressive strength of concrete are not less than *f*_0.8_ = 26,620.6 kN/m^2^, *f*_1.0 _= 16,798.9 kN/m^2^, *f*_1.2_ = 11,472.4 kN/m^2^ and *f*_1.4 _= 8267 kN/m^2^, respectively. It can be seen from the standard value of concrete axial compressive strength that when the width of the support body is 1.0 m, 1.2 m and 1.4 m, and the concrete strength is C40, C30 and C20, the wall fulfils the roadside support requirements. After integrating theoretical calculations and field practice, the flexible moulded concrete with a thickness of 1.2 m and a grade of C30 was selected for this practical study.

- Checking the calculation of the stability of the flexible wall

The stability checking calculation includes gangue side pressure calculation, overturning stability checking calculation and sliding stability checking calculation, among others. According to the on-the-spot investigation, the relevant parameters of formulas (1)–(3) can be obtained as follows: *γ *= 22 kN/m^3^, H_1 _= 2.2 m, *φ *= 30°, G = 48.4 kN/m, *a* = 0.4 m, *b* = 0.8 m, *α *= 42°, *z* = 0.8 m and *μ* = 0.3. The lateral pressure of the gangue *E*_*a*_ = 17.7 kN/m can be obtained through calculations. If *K*_t _= 2.42, the minimum anti-overturning safety factor is 1.5. If *K*_*a *_= 1.57, the minimum safety factor of the anti-sliding stability is 1.6.

The abovementioned stability checking calculation is conducted while considering the worst conditions. To prevent possible slips in the wall, steel bars will be planted in the wall roof and floor design in the subsequent design to improve the safety factor of the anti-sliding stability considerably.

- checking the calculation of the wall shape

**Fig 6:**
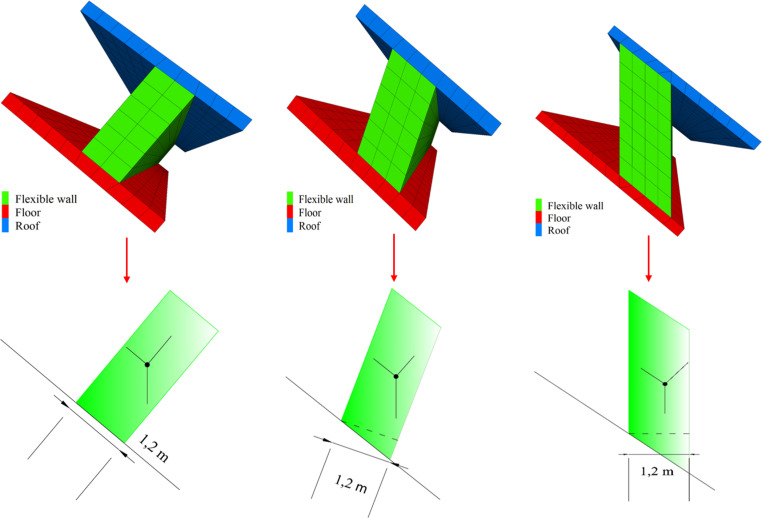
The shape analysis of flexible formwork wall. (a) Floor of vertical goaf; (b) Incline 20° to the goaf; (c) The floor of a vertical roadway.

Following the actual analysis and discussion on the spot, the authors finally decided to use three kinds of flexible formwork walls to analyse and demonstrate FFGER (Fig 6). The three shapes are: the wall is perpendicular to the floor of the goaf (Fig 6a), the wall is at an angle to the floor of the mining area (the angle of 20° is considered according to the on-site construction conditions) (Fig 6b), and the wall vertical roadway floor (Fig 6c).

**Fig 7:**
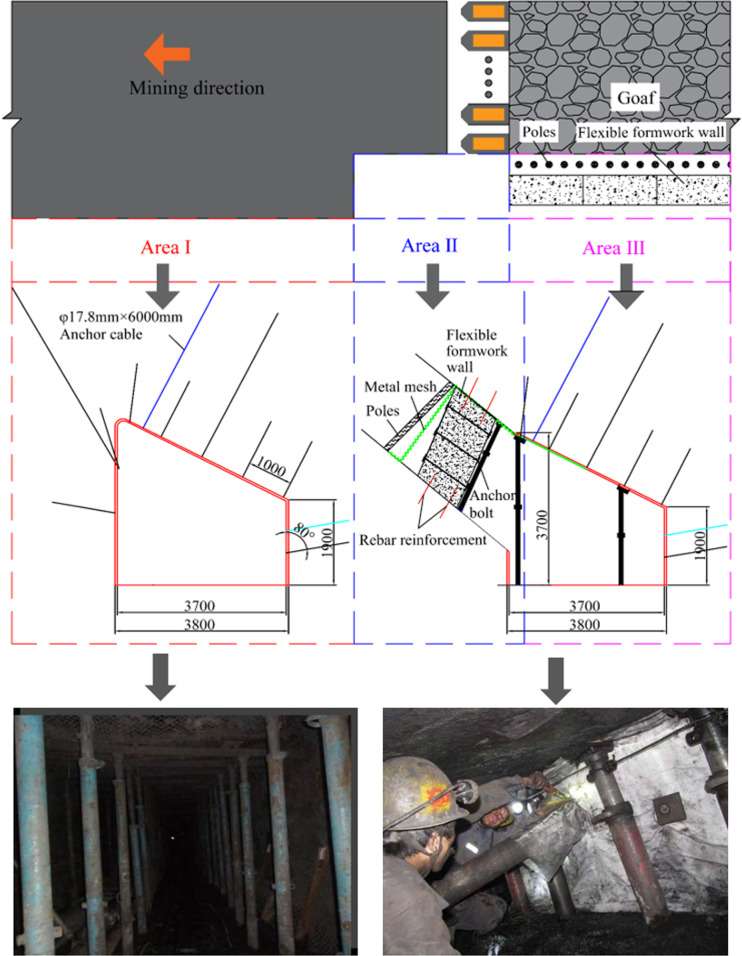
Design and practice.

Only the stress of the wall itself is considered in the checking calculation. Fig 7 demonstrates that with the change in the shape of the flexible formwork wall from the vertical goaf to the vertical roadway floor, the length of the long side of the wall continues to increase. Importantly, the length of the long side of the vertical roadway floor wall is the longest. The known formula for calculating the volume of a wall is described as follows:


D×T×W=V (7)

where *D* is the side length of the cross-section of the wall, m; *T* is the height of the cross-section, m; and *W* is the length of the direction of the flexible mould wall, m. Assuming that the volumes of the three states in Fig 6 are *V*_*a*_, *V*_*b*_ and *V*_*c*_, respectively, *V*_*a*_ < *V*_*b*_ < *V*_*c*_. As a result, with the change of the wall’s shape from the vertical goaf to the vertical roadway floor, the wall’s quality becomes larger and larger. The inverted slip occurs more easily, wherein the inverted slip risk of the floor wall of the vertical roadway is the greatest. However, when the wall is perpendicular to the floor of the goaf, the overall stress area on the side of the goaf is the smallest, and the possibility of the wall slip is greater. Therefore, considering various factors, such as the wall’s stress, the planting of steel bars on the roof and floor, and the conditions of hanging formwork in construction and economic investment, the authors finally decided to adopt the wall with an inclination of 20° to the goaf to perform the practice of FFGER.

### 4.2 Enhanced support design

Before the construction of FFGER, the roadway support must be strengthened. Using analysis and field practice, an anchor cable is added to the upper part of the roadway roof. The row distance between the reinforced anchor cables is 1.5 × 3 m, and the anchor cable specification is *Φ* = 17.8 × 6,000 mm. A bolt is added to bolster the coal side by hanging a net in the lower side of the roadway, which is consistent with the row distance between the original support. As a result, the supporting section is strengthened, as shown in Area I of Fig 7.

### 4.3 Roadside support design

Based on the theoretical calculation, the authors used the special-shaped flexible formwork concrete wall with a height of 1.8–2.4 m, length of 2 m or 3 m, width of 1.2 m, and strength of C30. The inclination of the wall was at a position of 20°, which facilitates the construction and simultaneously results in a better stress performance of the wall.

The impact protection system of the gangue for the retaining roadway along the goaf involves the metal mesh, wood point column, roof, and planting steel bar and protective plate of the bottom plate. The metal mesh is continuously laid before the hydraulic support in the working face to protect the roof and block the gangue. Meanwhile, a wooden point column is established in the lower part of the metal mesh, which can block the gangue and hang a flexible formwork with a diameter of 200 mm and a spacing of 0.5 m. A gangue baffle is installed at the shield beam of the support. The gangue plate is 1 m in height, 3 m in length and 4 cm in thickness. The gangue is fixed on the tail or base of the bracket by bolt or welding to block the gangue. In the pouring area of the wall next to the roadway, two rows of anchors are set up along the direction to plant steel bars. The bolt specification is *φ* = 18 × 1000 mm, and the distance between the rows is 0.5 × 0.5 m. The planting bar bolt exposed the roof and floor at 0.5 m. The concrete wall beside the roadway was pierced into the flexible formwork and poured into one to prevent the wall from slipping. This wall played the role of a blocking gangue. The supporting section of the roadside is shown in the Area II of Fig 7.

### 4.4 Design of flexible formwork concrete for coal solid waste

#### 4.4.1 Test materials and analyses.

**Fig 8:**
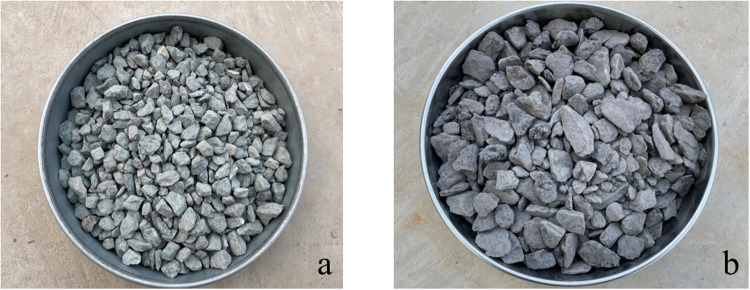
Coarse aggregate for test. (a) Stone; (b) Gangue.

The design of coal solid waste flexible formwork concrete of this institute is made of 42.5-grade cement, medium sand, stone, coal gangue and self-developed special admixture mixed with water according to a certain proportion. The final proportion of coal solid waste flexible formwork concrete comprises coal gangue while replacing some of the stone (the gangue was taken from the Daichiba Coal Mine). The gangue and stones are shown in Fig 8.

(1) Chemical composition

The data in Table 2 show that the primary chemical composition of the gangue is SiO_2_ and Al_2_O_3_, wherein the content of Fe_2_O_3_, K_2_O, CaO, MgO, TiO_2_, NaO, SO_3_ and other substances is relatively small. Regarding the chemical composition of gangue, the Dai Chi Ba coal mine gangue is suitable for coarse aggregate for concrete.

**Table 2:** Chemical composition of gangue and stone.

Chemical composition (%)	SiO_2_	Al_2_O_3_	Fe_2_O_3_	K_2_O	CaO	MgO	TiO_2_	NaO	SO_3_
gangue	59.55	19.81	7	4.39	4.06	1.73	1.25	0.9	0.6
stone	4.05	1.6	0.77	0.28	60.44	0.67	/	0.11	0.06

(2) Microscopic morphology

**Fig 9:**
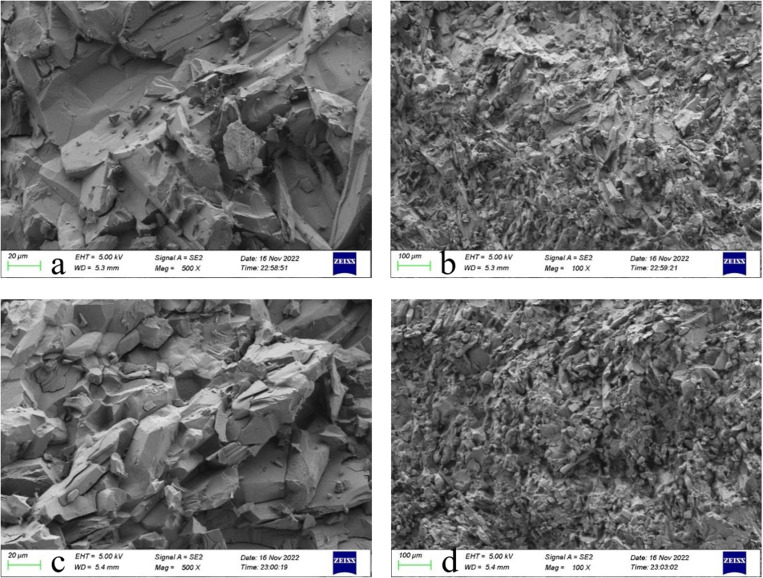
SEM electron microscope scan of gangue.

**Fig 10:**
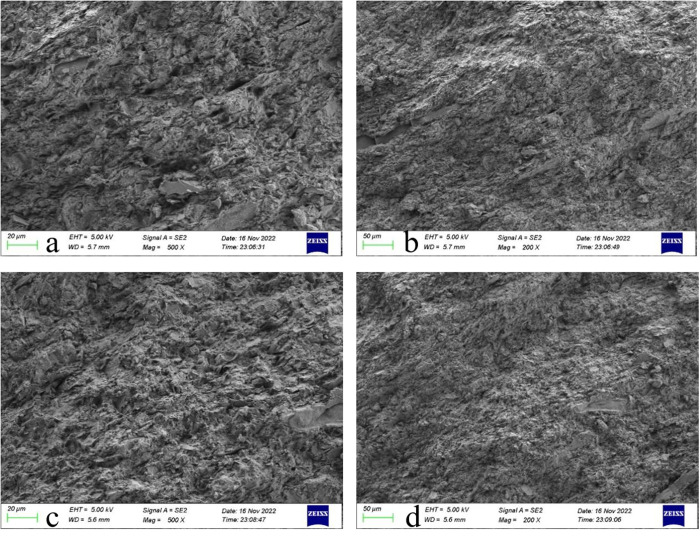
SEM electron microscope scan of stone.

The complexity of the coal gangue microstructure, characterised by higher density and three-dimensionality, significantly influences its physical properties, resulting in lower crush susceptibility, reduced mud generation, and enhanced firmness. The pilot study is displayed in Figs 9–10.

The SEM scanning situation demonstrates that the microscopic morphology of the stones is more three-dimensional compared to the gangue; the particles are interlaced and overlapped. The gangue microscopic morphology is relatively loose, mostly flaky and layered. This structure is easy to break, has low strength, is unstable, and is easily decomposed. Therefore, gangue concrete has lower strength and poorer concrete paste flow than stone concrete as a coarse aggregate for concrete formulation.

(3) Physical and mechanical properties

There are also differences in the physical and mechanical properties of gangue from different regions, rock formations and mining methods. Therefore, this paper analyses the feasibility of using coal gangue as coarse aggregate by investigating the physical and mechanical characteristics of coal gangue used as aggregate in the Dai Chi Ba coal mine (Particle gradation, crushing index, bulk density, apparent density, water content, water absorption, mud content and needle-flake particle content), and comparing the physical and mechanical properties of coal gangue with those of stones (Table 3).

**Table 3:** Test results of physical properties of gangue and stone.

Physical property	Stone	gangue
Grain composition (mm)	5 ~ 25	5 ~ 25
Crushing indicator (%)	10.6	21.3
Bulk density (kg/m^3^)	1533	1173
Apparent density (kg/m^3^)	2700	2328
Moisture content (%)	2%	4.4
Water absorption (%)	0.7	5.8
Mud content (%)	2.4	6.7
Needle-flake particle content (%)	3.1	5.9

Table 3 shows that despite having a large crushing index of coal gangue studied in this test, it fulfils the requirements for use as coarse aggregate. The apparent density is smaller than that of stones, proving that gangue has poor material features and is not dense enough. The content of needle and flake particles in gangue is known from the specification and meets the requirements for use as aggregate, but the mud content is larger. Therefore, the subsequent mass production of gangue concrete requires further sieving of the gangue. The water absorption rate of gangue is much larger than that of stone; hence, this should be fully considered in the test.

#### 4.4.2 Design of test ratios and analysis of results.

In this experiment, six sets of ratios will be designed using gangue to replace stones (0%, 20%, 40%, 60%, 80% and 100% by mass) (Table 4).

**Table 4:** Experimental proportioning design.

Number Matter	Cement (kg/m^3^)	Sand (kg/m^3^)	Stone (kg/m^3^)	gangue (kg/m^3^)	Water (kg/m^3^)	admixture (kg/m^3^)
1	650	700	800	0	300	0.8
2	650	700	640	160	300	0.8
3	650	700	480	320	300	0.8
4	650	700	320	480	300	0.8
5	650	700	160	640	300	0.8
6	650	700	0	800	300	0.8

(1) Slump and Extension Analysis

**Fig 11:**
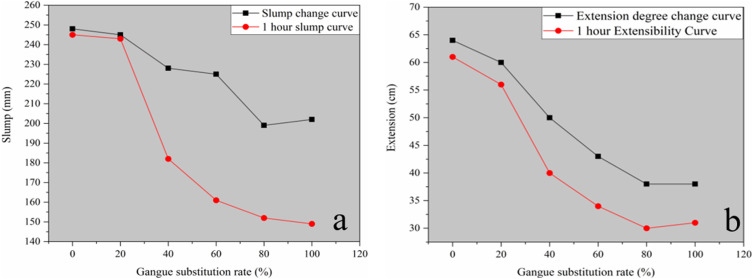
Analysis of test mix properties. (a) Slump change curve; (b) Extension degree change curve.

When the concrete flow has stopped, the slump of the concrete is measured with a slump scale. The final spreading diameter of the concrete is measured after spreading with a steel ruler. Measurements are performed in two directions perpendicular to each other (the maximum diameter and the direction perpendicular to the maximum diameter), and the average of the measured diameters is calculated, the results of which are shown in Fig 11.

The trend in Fig 11 shows that an increase in the gangue substitution rate causes the slump and ductility of concrete to decrease rapidly before levelling off. Among them, the decrease is the largest when the coal gangue substitution rate exceeds 40%. When the substitution rate exceeds 80%, it tends to be flat out. Fig 11 reflects that both slump and expansion suffered losses after 1 hour, and the degree of loss was significantly positively correlated with the coal gangue replacement rate. Among them, the loss is small when the substitution rate does not exceed 20%, but the loss of slump and expansion is the largest when the substitution rate reaches 40%. Notably, the loss tends to be flat when the substitution rate exceeds 80%.

(2) Mechanical property analysis

After adhering to the test programme designed in Table 4, the cubic compressive strength and the axial compressive strength for 3 d, 7 d and 28 d were determined (Table 5).

**Table 5:** Compressive strength test results.

Number	Cubic compressive strength (MPa)			Axial compressive strength (MPa)
	3d	7d	28d	28d
1	17.2	29.7	48.5	44.3
2	19.8	23.1	35.4	31.3
3	17.8	22.3	35	24.3
4	14.8	19.6	31.7	23.8
5	16.1	21.6	32.8	23.2
6	16.3	21.2	27.7	23

**Fig 12:**
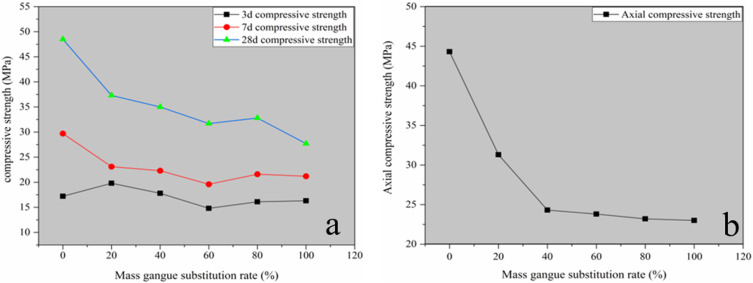
Analysis of concrete compressive strength tests. (a) Cubic compressive strength; (b) Axial compressive strength.

Fig 12 can be obtained from the compressive strength test data statistics. The trend in Fig 12A demonstrates that the increase in the gangue substitution rate decreases the concrete’s strength. However, removing the 100% substitution rate, the 28-d strength of concrete for the rest of the ratios is greater than 30 MPa. The compressive strength at three days differed from the alterations in the compressive strength at 7 and 28 days, and the compressive strength at 3 days generally maintained a stable trend of change, with an increase occurring when the substitution rate reached 20%. This phenomenon is attributed to the gangue water absorption rate being greater than the stone; therefore, in the early period, different substitution rate strength attenuation is smaller due to the gangue water absorption rate caused by the high water-cement ratio of concrete, which leads to the faster growth of the three-day compressive strength. The variation in the axial compressive strength in Fig 12B is generally consistent with the law that the axial compressive strength of ordinary concrete is much less than the compressive strength of cubes and is therefore not analysed further here.

**Fig 13:**
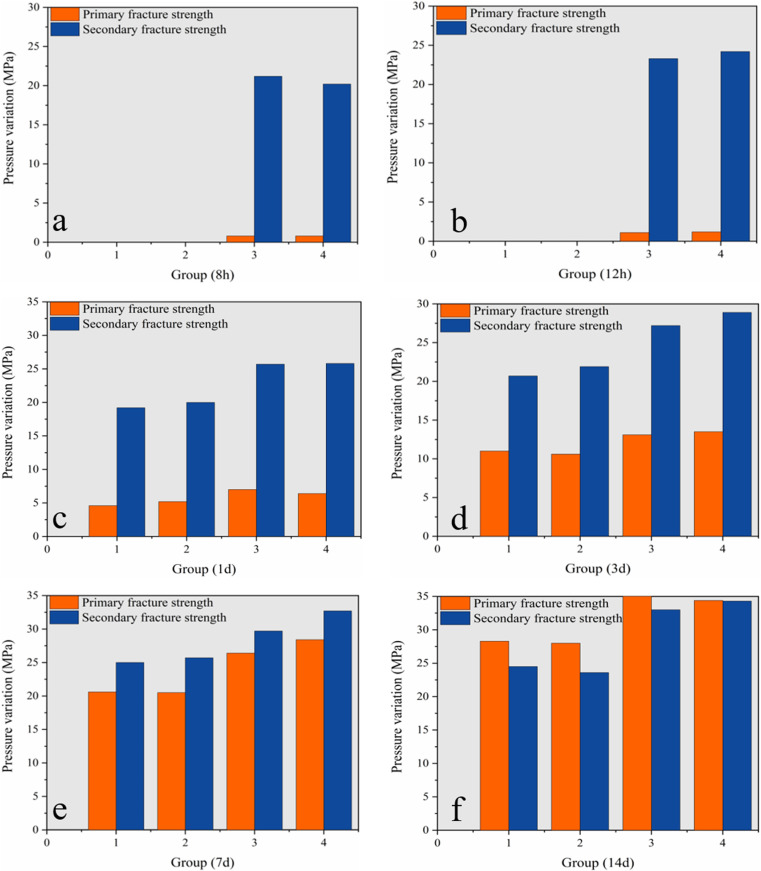
Results of compressive strength test.

#### 4.4.3 Final concrete mix ratio.

Optimisation through experimental analysis resulted in the finalised formulation (Table 6).

**Table 6:** Mix ratio of C30 flexible concrete.

Material	Cement	Sand	Stone	Gangue	Water	Admixture
Mass (kg)	650	700	480	320	300	0.8

#### 4.4.4 Mechanical test and analysis.

- Test arrangement

This coal solid waste flexible formwork concrete cube compressive strength test was performed using cubic specimens (150 × 150 × 150 mm). A total of 84 specimens with a concrete strength of C30 were divided into groups of 3 and were used to prepare the standard group (SG) and flexible formwork group (FFG). The SG plain concrete specimens were tested for strength at ages 1 d, 3 d, 5 d, 7 d, 14 d and 28 d. The age groups were grouped and numbered 1 and 2. In the FFG, flexible form concrete specimens were tested for strength at ages of 8 h, 12 h, 1 d, 3 d, 5 d, 7 d, 14 d and 28 d. The age groups were divided and numbered 1 and 2.

- Compression strength test

Each group contained three specimens in the compressive strength test, and the average values were accurate to 0.1 MPa. The SG specimens were tested for strength at ages of 1 d, 3 d, 5 d, 7 d and 14 d. Coal solid waste FFG specimens were tested for strength at ages of 8 h, 12 h, 1 d, 3 d, 5 d, 7 d and 14 d. The measurement of pressure to produce cracks is called primary compression cracking, and this pressure is continuously maintained. This pressure was to be kept for 28 d after the measurement of its compressive strength again, which is called secondary compression cracking.

Ordinary concrete must be cured for at least one day before it can be demoulded. In contrast, flexible concrete can be demoulded and tested for compressive strength at 8 h and 12 h due to the encasement of the flexible formwork.

- Strength analysis of primary fracturing.

**Fig 14:**
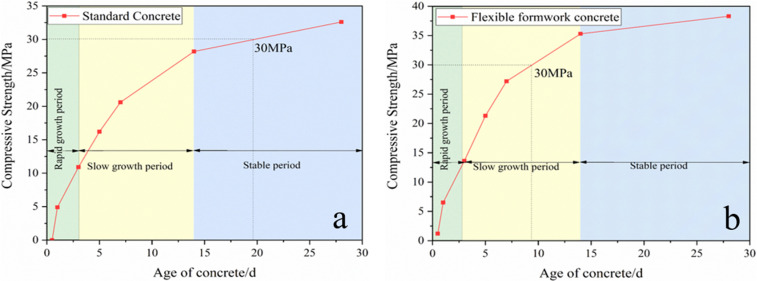
Comparative age-growth curves.

According to the test results (as shown in Fig 13), the growth curve of the strength (average value of primary fracturing strength) at each age of the standard group and flexible module group concrete can be obtained, as shown in Fig 14.

Fig 14 demonstrates that the strength of coal solid waste flexible formwork concrete at all ages is higher than the strength of the standard set of concrete with the progression of time. The strength growth of FFG concrete for 1–7 days is faster, and the strength for approximately 7 days can reach the design value.

#### 4.4.5 Residual strength of flexible form concrete.

The primary mechanical characteristics of concrete during compression are demonstrated as follows:

- Damage to the concrete begins with an original defect, a crack or microcrack, that existed before the concrete was loaded.
- Microcracks exist within concrete under external loading, whether located at the coarse aggregate-cement interface or in the cementitious substrate. The expansion of microcracks leads to the deterioration of the concrete material due to stress concentration.
- When the concrete is subjected to axial loading, tensile stresses and strains will be generated in its transverse direction, destroying the material when it reaches its tensile limit.
- The elastic and inelastic deformation of concrete under axial compressive loading should be analysed in terms of longitudinal and transverse stress-strain relationship curves and volume expansion of concrete.

The test data display that after the first fracturing of each age, although there were cracks, the compressive strength of the secondary fracturing was maintained and had not decreased. Instead, the strength was considerably improved. It can be observed that ordinary concrete already has a certain residual strength, and the flexible formwork concrete was outside the package of flexible formwork. Formwork bags exert a positive effect on the residual stresses around the pressure. This phenomenon shows that the flexible formwork concrete has a large residual strength.

In flexible formwork concrete structures, the lateral compressive stresses on the concrete are passive and increase with the vertical pressure. Flexible formwork and concrete deformation were more coordinated during the early loading stage. Subsequently, it can be inferred that the concrete was in a unidirectional force state, and the restraining effect of flexible formwork did not work. With the increase in the vertical load, the transverse deformation coefficient of flexible formwork and concrete will continue to increase. Suppose the transverse deformation coefficient of concrete exceeds that of the flexible formwork. In that case, the flexible formwork will constrain the core concrete, and the passive lateral pressure will occur and rise with the increase in the vertical pressure.

#### 4.4.6 Healing capacity and mechanism analysis.

**Fig 15:**
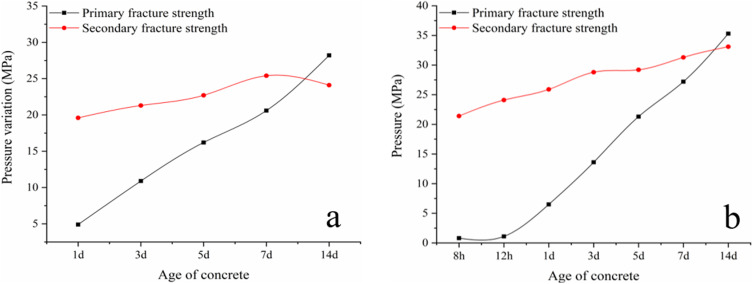
Compressive strength comparison. (a) Standard group; (b) Flexible formwork group.

The first crack strength and second crack strength of plain concrete and flexible formwork concrete were analysed, respectively, and the test results are demonstrated in Fig 15.

The test findings reveal that the repair ability of the flexible formwork concrete is superior to that of regular concrete because the specimen is in the continuous loading process due to the specimen in the microcrack opening, expansion, penetration and other observed phenomena. Hence, the specimen pore space continues to grow, which is prompted by its increased macroscopic manifestation of the volume. The peripheral formwork restricts the expansion capacity of flexible formwork concrete, and an increase in the constraint will force the expansion to diminish. The composite concrete developed by the flexible formwork and concrete heals more readily.

The hydration of cement begins after mixing with water, and the hydration reaction of cement gradually moves deep into the inner layer from the surface of the particles. This action is rapid at the beginning, but later, due to the generation of a gel film around the cement particles, water ingress becomes increasingly challenging. Therefore, hydration becomes slower and slower. In fact, coarser cement particles will not be fully hydrated internally for a long time. The hardened cement stone, therefore, comprises crystals, colloids, incompletely hydrated cement particles, free water and pores. At different ages in the hardening process, the proportion of each component in the cement stone is different, directly impacting the strength and other properties. When cracks appear in concrete, the cracks can gradually heal, primarily due to the following factors:

- In general, cement particles with a particle size greater than 10 *μ*m account for approximately 50% of the cement, wherein the maximum particle size can reach 100 *μ*m. Because the hydration depth of cement is 5–9 *μ*m in 9 months, the unhydrated cement still exists in concrete even if it is cured for a long time. During the early hardening stage, unhydrated cement particles account for a large proportion. Therefore, once the crack occurs, water seeps through the crack and the surrounding pores, prompting the unhydrated cement to continue to hydrate. The hydration products gradually fill the fracture surface to restore the compressive strength.
- Calcium hydroxide crystals are present in the hydration products of cement [34,35]. When the crystals react with carbon dioxide in the surrounding air and water, they form carbonates, creating carbonate and calcium hydroxide crystals in the cracks, which accumulate and fill the cracks. The mechanical adhesion they produce is used to supplement the chemical cohesion between one crystal and another crystal and between the paste surface wrapped by the crystal and aggregate so that the compressive strength of the specimen can be restored.
- Because the concrete with cracks is cured under moisture, the cement that stopped hydration is rehydrated due to water infiltration near the cracks. The hydration products are moved to the crack surface through capillary movement, thus accelerating the healing of the crack surface.

### 4.5 Engineering practice

**Fig 16:**
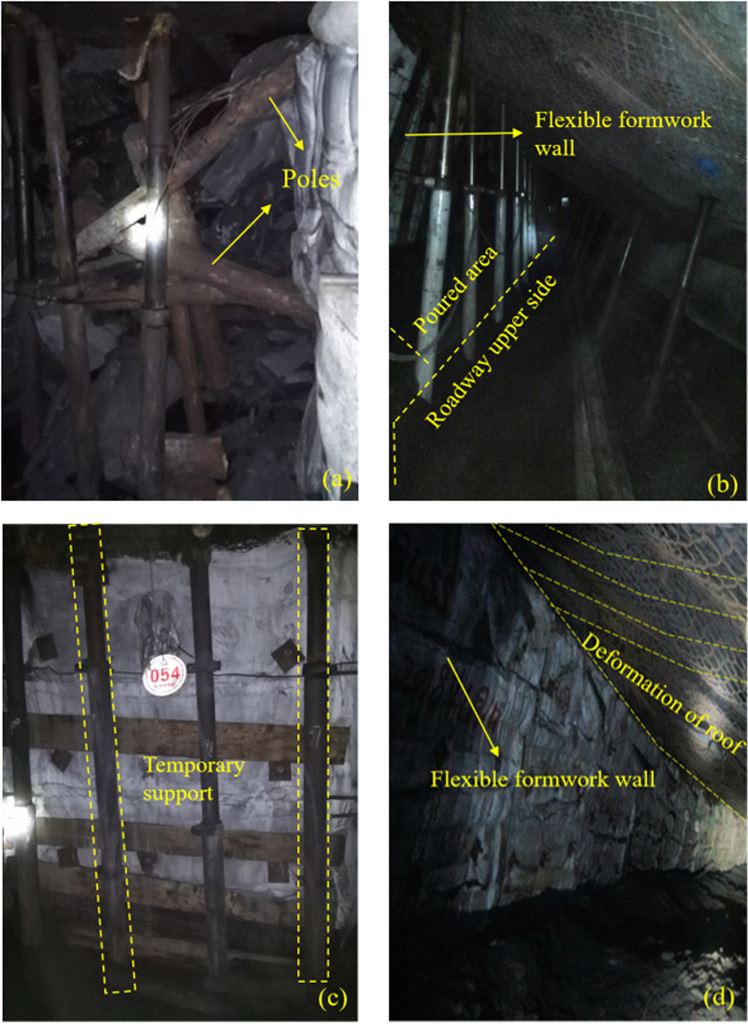
Field effect. (a) Roadway deformation monitoring; (b) Wall stress monitoring.

**Fig 17:**
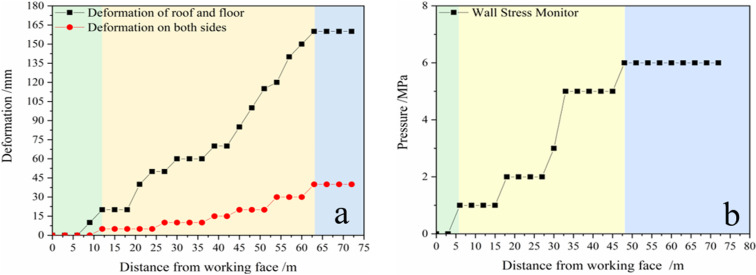
Engineering practice monitoring results.

The engineering effect revealed that the design and application of the technology of FFGER at high-angle and medium-thick coal seams was satisfactory, and the roadway deformation was finally stable after mining. Therefore, this technology can be used in industry, and the relevant engineering practice is shown in Fig 16. This technology has been applied for the first time to a medium-thickness coal seam with a large dip angle in this study. Therefore, continuous monitoring and optimisation of this technology is required. During engineering practice, on-site roadway deformation and roof and floor stress monitoring are performed simultaneously. Displacement sensors are used for roadway deformation monitoring, and the wall pressure sensors are used for roof and floor stress monitoring. The first group of sensors is arranged in the roadway 30 m away from the working face cut. The on-site monitoring data are demonstrated in Fig 17. The analysis data reveal that the wall pressure reaches the maximum when the gob-side entry is 80 meters from the cut-off, and the coal wall on the lower wall side deforms substantially. The roof movement reaches the maximum within the range of 60–65 m of the lagging working face. However, overall, the tunnel pressure after the roadway retention does not appear apparent, and the filling body does not deform significantly.

**T**he engineering effect demonstrates that the impact of FFGER in a medium-thick coal seam with a high dip angle in the Daichiba Coal Mine is superior in the whole stage (Fig 16). The successful application of this technology further enhances the technical experience of non-pillar mining in the Daichiba Coal Mine.

## 5 Conclusion

The application of this study culminated in the following conclusions.

- According to the features of large inclined and medium-thick coal seams, this study presents a formula for the stability inspection of flexible formwork walls and systematically introduces FFGER.
- Based on the technical principle of FFGER, a mechanical model of the surrounding rock of a medium-thickness coal seam with a large inclination angle in the Daichiba Coal Mine and a formula for calculating the bearing capacity of the flexible formwork wall were established. A self-developed special-shaped flexible formwork concrete wall with a width of 1.2 m, a strength of C30 and an inclination angle of 20° was selected to implement GER.
- According to full consideration of the engineering background and combined with the preliminary research and observation, the roadway strengthening support along the mining area of No. 31342 working face and the roadway side support was determined.
- Using local materials, it is proposed that coal solid waste (gangue) be mined to replace the part of the stone to perform experimental research on coal solid waste flexible formwork concrete. The final proportion is: cement = 650 kg/m^3^, sand = 700 kg/m^3^, natural gravel = 480 kg/m^3^, gangue = 320 kg/m^3^, water = 300 kg/m^3^ and additive = 0.8 kg/m^3^.
- The engineering practice results show that the design of the flexible formwork retaining roadway along the goaf in the No. 31342 working face is suitable and optimal, and the application effect is excellent. This research can provide useful reference for problems encountered in large and even steeply inclined coal seams.

## Supporting information

S1 Data Raw data. (ZIP)
