# Supplementary figures and images for: Research analytics on the applications of flexible formwork gob-side entry retention technology in medium-thickness coal seams with large inclination angles
Source: PLoS One. 2025 May 12;20(5):e0323337. doi: 10.1371/journal.pone.0323337 (PMC12068642; doi:10.1371/journal.pone.0323337)

## Working face production, gob-side entry retaining construction process

## work requirement

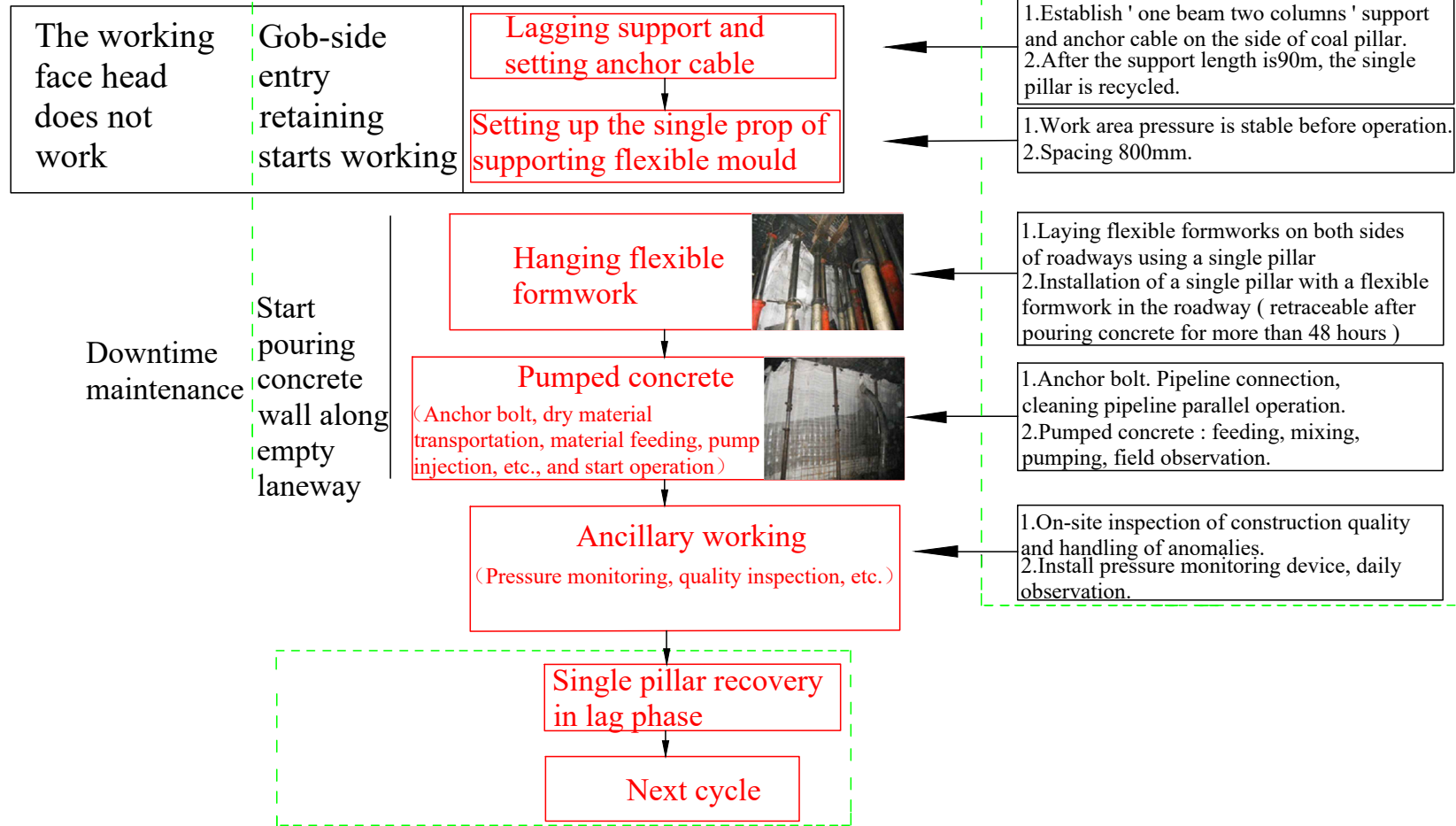

Supplement: S1 Data — (ZIP) [file pone.0323337.s001.zip › Raw data/流程.pdf]

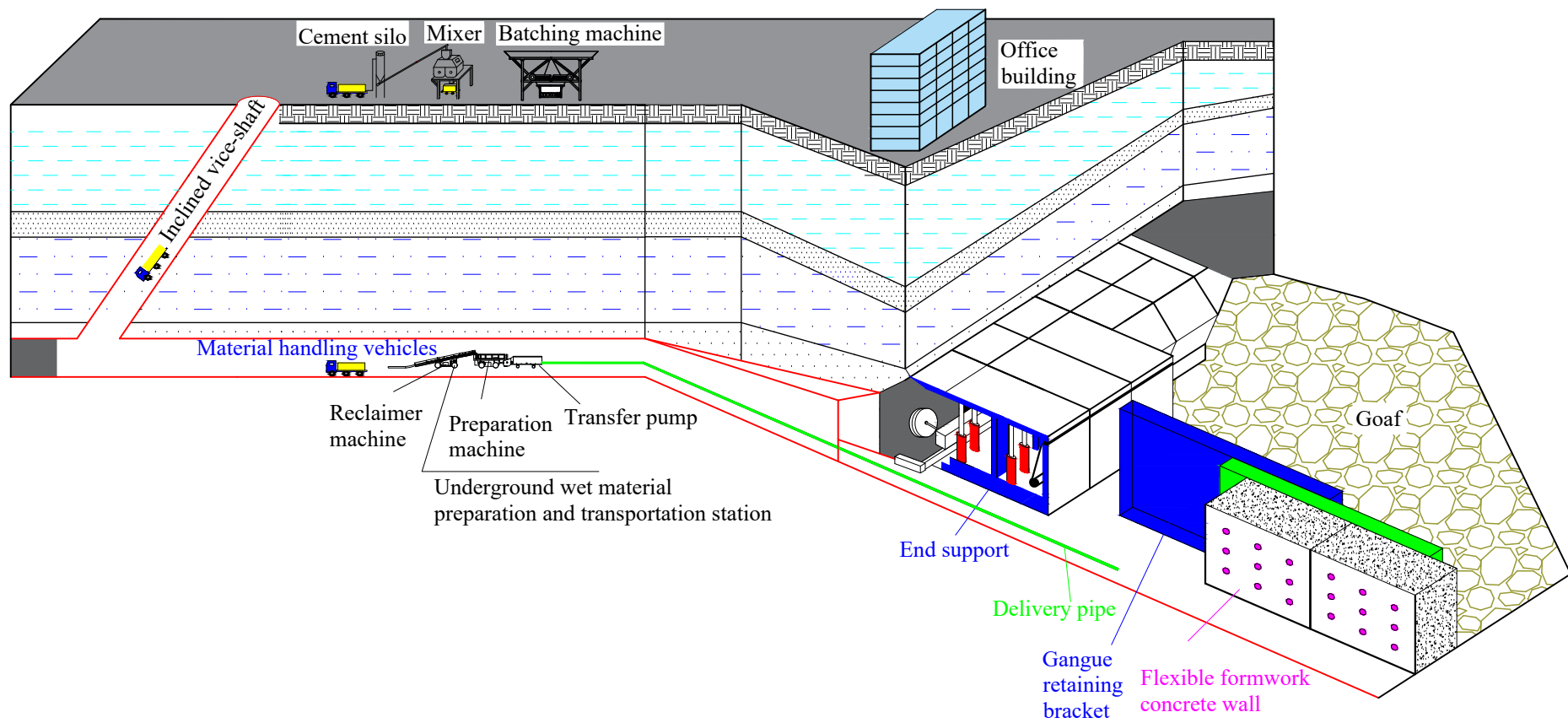

Supplement: S1 Data — (ZIP) [file pone.0323337.s001.zip › Raw data/柔模支护施工工艺.pdf]

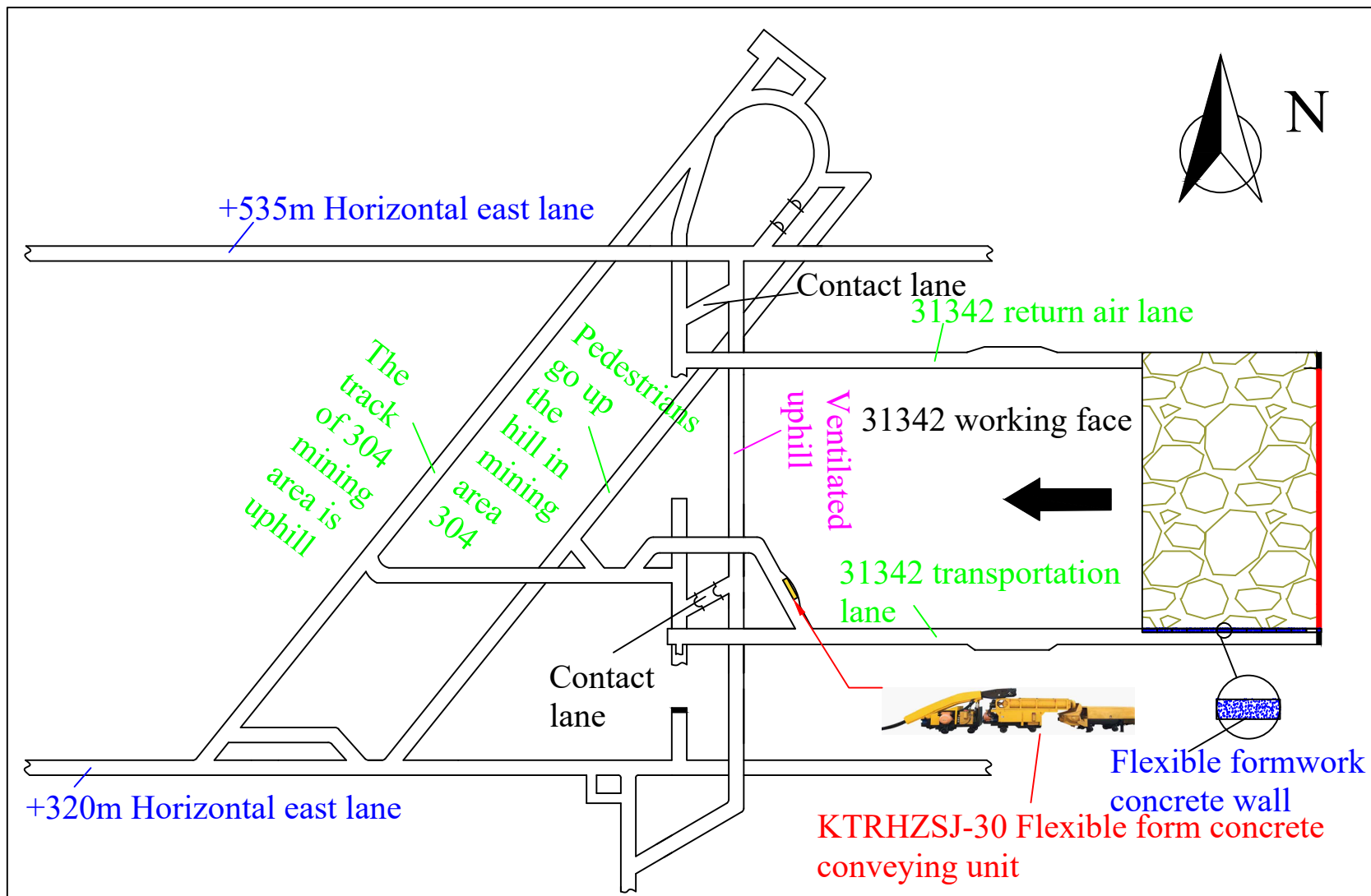

Supplement: S1 Data — (ZIP) [file pone.0323337.s001.zip › Raw data/工作面布置图.pdf]
